# Mechanical circulatory support as bridge therapy for heart transplant: case series report

**DOI:** 10.1186/s13104-018-3515-2

**Published:** 2018-07-03

**Authors:** Javier D. Garzon-Rodriguez, Carlos Obando-Lopez, Manuel Giraldo-Grueso, Nestor Sandoval-Reyes, Jaime Camacho, Juan P. Umaña

**Affiliations:** 1 Universidad del Rosario, Fundacion Cardioinfantil-Instituto de Cardiología, Bogotá, Colombia; 2 Cardiac Surgery Department, Fundación Cardioinfantil-Instituto de Cardiología, Bogotá, Colombia; 3 Vascular Function Research Laboratory, Fundación Cardioinfantil-Instituto de Cardiología, Calle 163 A número 13B-60, 111831 Bogotá, Colombia

**Keywords:** Heart-assist devices, Extracorporeal membrane oxygenation, Heart transplantation, Myocardial ischemia

## Abstract

**Background:**

Mechanical circulatory support (MCS) represents an effective urgent therapy for patients with cardiac arrest or end-stage cardiac failure. However, its use in developing countries as a bridge therapy remains controversial due to costs and limited duration. This study presents five patients who underwent MSC as bridge therapy for heart transplantation in a developing country.

**Case presentation:**

We present five patients who underwent MCS as bridge therapy for heart transplant between 2010 and 2015 at Fundación Cardioinfantil-Instituto de Cardiología. Four were male, median age was 36 (23–50) years. One patient had an ischemic cardiomyopathy, one a lymphocytic myocarditis, two had electrical storms (recurrent ventricular tachycardia) and one an ischemic cardiomyopathy with an electrical storm. Extracorporeal life support (ECLS) was used in three patients, left ventricular assistance in one, and double ventricular assistance in one (Levitronix^®^ Centrimag^®^). Median assistance time was 8 (2.5–13) days. Due to the inability of cardiopulmonary bypass weaning, two patients required ECLS after transplant. One patient died in the intensive care unit due to type I graft rejection. Endpoints assessed were 30-day mortality, duration of bridge therapy and complications related to MCS. Patients that died on ECLS, or were successfully weaned off ECLS were not included in this study.

**Conclusions:**

MCS is often the only option of support for critically ill patients waiting for a heart transplant and could be considered as a short-term bridge therapy.

## Background

The increase of life expectancy and ischemic heart disease within the years has created long heart transplant waiting lists. According to the United Network for Organ Sharing, more than 3000 hearts per year are needed to meet the waiting list demand in the United States; however, less than 2000 heart donors per year are reported [1]. In Colombia the median waiting list time for a heart transplant was 59 (1–606) days in 2014 [2]. The use of extracorporeal life support (ECLS) and ventricular assist devices as a bridge therapy for a heart transplant has been described as a feasible alternative [1–4]. However, the cost of the therapy, the success rate and the limited duration of ECLS remain as subjects of concern [1–4]. We present five cases of mechanical circulatory support (MSC) as a bridge therapy for a heart transplant and the short-term results.

## Case presentation

From January of 2010 to December 2015, five patients underwent ECLS or ventricular assist devices as bridge therapy for heart transplantation at Fundación Cardioinfantil-Instituto de Cardiología, Bogotá-Colombia. Patients that died on ECLS, or were successfully weaned off ECLS are not presented. The ventricular assist device used was the Levinotrix^®^ CentriMag^®^ (levitronix LLC; waltham, mass).

The indications for MCS were: Ischemic cardiomyopathy, lymphocytic myocarditis, and an electrical storm (recurrent ventricular tachycardia). The interagency registry for mechanically assisted circulatory support (INTERMACS) was assessed in all patients. The primary endpoints were 30-day mortality, duration of bridge therapy and complications related to MCS.

Four patients were male. All patients were New York heart association class IV. The median left ventricular ejection fraction was 19% (15–20). One patient had an ischemic cardiomyopathy, one a lymphocytic myocarditis, two had electrical storms and one an ischemic cardiomyopathy with an electrical storm (Table 1).

**Table 1:** Patient characteristics

Variables	Case 1	Case 2	Case 3	Case 4	Case 5
Demographic and clinical					
Age (mean, SD)	50	21	51	25	36
Male	No	Yes	Yes	Yes	Yes
Hypertension	Yes	No	Yes	No	Yes
Chronic kidney disease	No	No	No	Yes	Yes
Diabetes mellitus	No	No	No	No	Yes
LVEF (%)	15	19	20	21	16
INTERMACS 1	Yes	Yes	Yes	Yes	No
INTERMACS 2	No	No	No	No	Yes
Myocarditis	No	No	No	Yes	No
Electrical storm	Yes	Yes	No	No	No
Ischemic cardiomyopathy	Yes	No	Yes	No	Yes
MCS support					
ECLS	Yes	No	No	Yes	Yes
Ventricular assistance	No	Yes	Yes	No	No
Vascular complications	Yes	No	Yes	No	No
Time (days)	8	2	15	3	11
Post-transplant					
Graft rejection	No	No	No	No	Yes
Reoperation for bleeding	No	No	No	Yes	No
Mortality	No	No	No	No	Yes
Stroke	Yes	No	No	No	No
ECLS	Yes	No	No	No	Yes
Intra-aortic balloon pump	Yes	No	Yes	Yes	Yes

Before MCS, three patients required inotropic support; four patients had an intra-aortic balloon pump. One patient had mechanical ventilatory support and three had organ dysfunction (acute kidney failure). All patients underwent catheter ablation therapy due to rhythm abnormalities. Four patients were INTERMACS 1 and one INTERMACS 2. One patient presented transit ischemic attack (right-side hemiparesia that disappear within 1 h, without medical therapy) (Table 1).

During MCS, one patient required left ventricular assistance, two ECLS, and one biventricular assistance. The median time of MCS prior to heart transplantation was 8 (2.5–13) days. Two patients had vascular complications during MCS, one developed leg ischemia; the other one required a surgical exploration due to bleeding at the insertion site of the femoral cannulae (Table 1).

After the heart transplant, four patients continued with an intra-aortic balloon pump, and two required ECLS due to the inability of cardiopulmonary bypass weaning. Two patients developed a pericardial effusion; both of them required a percutaneous drainage. One patient developed a hemopericardium that was managed through a sternotomy. One patient died in the intensive care unit due to type 1 graft rejection and multiple organ dysfunctions (Table 1).

## Discussion and conclusions

MCS is a useful alternative for the management of critically ill patients with cardiogenic shock, refractory cardiac failure or potentially fatal arrhythmias [5–11]. In Latin America, there are no publications describing the use of ECLS and ventricular assist devices as a bridge therapy for heart transplantation, and adult patients are rarely bridged to transplantation due to difficulties in the urgent medical evaluation. Therefore this study contributes to an emerging field and could indicate ECLS and ventricular assist devices are a feasible bridge for a heart transplant.

All the patients we described received MCS as a bridge for transplant. Waiting time for organ availability was short since the average duration of the bridge therapy was 8 days. Some studies described the need for at least 3 weeks of ECLS before transplant [3]. However, poor outcomes are reported with prolonged use of ECLS, the frequency of bleeding, infection or embolism begins to rise after the first 7 days [4]. In our series, all patients were listed as high-urgency.

Recent studies have shown good results after a heart transplant in patients with preoperative ECLS [8–11]. Pre-existing organ dysfunction elevated blood urea, and liver function are significant predictors of death. Therefore if the patient has multiple organ failures the bridge therapy should not be considered [4, 12].

The prognosis after heart transplant seems to be superior in patients with INTERMACS 1, which underwent ECLS at an early stage [13]. In our series, we showed that all patients INTERMACS 1 accomplished hemodynamic stabilization during ECLS therapy that enabled them to achieve a successful heart transplant.

Patients in our series, were young (median age 36 years) had a poor functional class, a low left ventricular ejection fraction (median 19%) and biventricular failure with pulmonary edema. Barth and colleagues [3] reported a cohort of 8 patients with bridge therapy, the mean left ventricular ejection fraction was 17.5%, the mean age was 49 years and they also had a poor functional class.

Ischemic cardiomyopathy is the most common indication for a heart transplant [3]. In our series we found a high frequency of electric storms, these patients received a conservative inotropic management and underwent ECLS due to the high risk of sudden death. Two patients had vascular complications during MCS, one developed leg ischemia; the other one required a surgical exploration due to bleeding at the insertion site of the femoral cannulae. In both patients, the cannulae was adjusted and no motor deficits were observed.

In our series, we found a high incidence of bleeding reoperations. Three patients developed cardiac tamponade secondary to hemopericardium or pericardial effusion, which was managed with pericardiocentesis or a median sternotomy. This is higher than the previously reported series, which describes 28% of bleeding reoperations [8]. Ranjit and colleagues [14] reported a 4% frequency of infectious complications, although our series have only five patients, it is worth noting that the freedom from infectious complication was 100%.

Only one patient died after heart transplant as a result of a type 1 graft rejection which evolved to multisystemic organ failure. This condition is associated with a high mortality rate (almost 80%) despite optimal treatment, or retransplantation [2].

MCS is often the only option of support for critically ill patients (refractory heart failure, cardiogenic shock or electric storm) needing a heart transplant. We suggest considering ECLS and ventricular assist devices as a short-term bridge therapy, since the mechanic support becomes a real alternative due to hemodynamic stabilization and gives time to find a heart donor. However, patients in ECLS have more complications, especially with increasing duration of use.

## Abbreviations

ECLS: extracorporeal life support
INTERMACS: the interagency registry for mechanically assisted circulatory support
MCS: mechanical circulatory support
